# Participation of the Classical Speech Areas in Auditory Long-Term Memory

**DOI:** 10.1371/journal.pone.0119472

**Published:** 2015-03-27

**Authors:** Anke Ninija Karabanov, Rainer Paine, Chi Chao Chao, Katrin Schulze, Brian Scott, Mark Hallett, Mortimer Mishkin

**Affiliations:** 1 National Institute of Mental Health, Bethesda, Maryland, United Sates of America; 2 Danish Research Center for Magnetic Resonance, Hvidovre, Denmark; 3 National Institute of Neurological Disorders and Stroke, Bethesda, Maryland, United Sates of America; 4 Institute of Child Health, University College London, London, United Kingdom; 5 Department of Neurology, National Taiwan University Hospital, Taipei, Taiwan; IIT—Italian Institute of Technology, ITALY

## Abstract

Accumulating evidence suggests that storing speech sounds requires transposing rapidly fluctuating sound waves into more easily encoded oromotor sequences. If so, then the classical speech areas in the caudalmost portion of the temporal gyrus (pSTG) and in the inferior frontal gyrus (IFG) may be critical for performing this acoustic-oromotor transposition. We tested this proposal by applying repetitive transcranial magnetic stimulation (rTMS) to each of these left-hemisphere loci, as well as to a nonspeech locus, while participants listened to pseudowords. After 5 minutes these stimuli were re-presented together with new ones in a recognition test. Compared to control-site stimulation, pSTG stimulation produced a highly significant increase in recognition error rate, without affecting reaction time. By contrast, IFG stimulation led only to a weak, non-significant, trend toward recognition memory impairment. Importantly, the impairment after pSTG stimulation was not due to interference with perception, since the same stimulation failed to affect pseudoword discrimination examined with short interstimulus intervals. Our findings suggest that pSTG is essential for transforming speech sounds into stored motor plans for reproducing the sound. Whether or not the IFG also plays a role in speech-sound recognition could not be determined from the present results.

## Introduction

Speech sounds fluctuate at high, millisecond speeds, and it appears that integrating and storing such rapidly varying signals cannot be carried out by the auditory system alone. This supposition is based on the evidence that storing new speech sounds requires reproducing or mimicking those sounds [1], and it is likely that the same applies to storing new melodies. That mimicking is a prerequisite for laying down an auditory memory is suggested by two related findings: First, auditory stimuli that humans have great difficulty mimicking, such as reversed words, are ones that humans have great difficulty recognizing a few minutes after hearing them [1]; and second, mammals such as dogs and monkeys that cannot mimic their conspecifics' vocalizations, unlike marine mammals and songbirds that can do so, seem to be devoid of auditory recognition memory [1–4]. These findings imply that the formation of long-term auditory memories requires the assistance of the motor system, and this, in turn, suggests that in humans, the arcuate fasciculus, a bidirectional pathway that directly connects the auditory and oromotor systems, with end stations in the posterior portion of the superior temporal gyrus (pSTG) and the inferior frontal gyrus (IFG) [5,6], plays an essential role in storing the central representations of acoustic stimuli.

In the influential working memory model of Baddeley and Hitch [7], verbal information is processed by a phonological loop, which is further subdivided into a passive storage component (phonological storage) and an active rehearsal mechanism (articulatory rehearsal process). Whereas the passive storage is assumed to store auditory information only for a few seconds [8], the articulatory rehearsal process can maintain information for longer time spans [9].

There is converging evidence to support the notion that articulatory rehearsal is supported by subvocal speech: (i) participants show a greater memory span [10] and superior recognition accuracy [11] for short compared to long words; (ii) the articulatory rehearsal process can be interrupted by preventing internal rehearsal of verbal material [9,12–14]; and (iii) neuroimaging studies have reported that subvocal rehearsal of verbal material engages motor-related areas [9,15–20]. Thus, participants might use their ability to produce speech in order to convert the aurally presented verbal information into internally rehearseable motor representations or sensorimotor codes [16,17,19].

Whereas Baddeley’s working memory model recognizes the importance of subvocal articulation (or rather verbalization) to refresh or retrieve (verbal) memory traces, it does not make any claims about the form in which auditory memory traces are stored in long-term memory. The idea that the motor system is pivotal in the formation of auditory long-term memories expands the importance of articulation to long-term storage of a wider array of auditory stimuli, especially when these stimuli cannot be attached to a semantic association.

Indeed, Hickok and Poeppel’s well known dual-stream model of speech processing [21] can be expanded to explain more general processes of auditory long-term memory: In particular, the dorsal stream, which follows the arcuate fasciculus and maps acoustic speech signals onto frontal articulatory networks, might play an important role in auditory and verbal working memory. The dorsal stream connects the pSTG (referred to as Spt by [21]) with the articulatory motor networks in and around the IFG.

Several neuroimaging studies suggest a role of the temporo-parietal areas in and around the pSTG as a sensorimotor interface: Left parietal-temporal areas increase activity during the delay period of verbal working memory tasks, independent of the modality of the presented stimuli [17,22] and are activated not only by a wide range of auditory stimuli (speech and music) but also by oromotor behavior (covert speech/humming) [17,23].

The left IFG, often referred to as Broca’s area, has also been implicated in verbal working memory [9,15,18,24–26], as well as in tonal (auditory) working memory [16,17,19]. The supposition that Broca’s area participates in verbal working memory is further strengthened by evidence from repetitive TMS (rTMS) studies confirming this area's necessary participation in phonological and verbal working memory [27,28]. Imaging data also suggests that the IFG is especially important during auditory-verbal long-term memory: Buchsbaum and co-workers [29] could show that activity in the IFG increases as a function of increasing time delay between word encoding and recognition whereas activity in the temporo-parietal cortex showed the opposite pattern.

Our goal in the current study was to further investigate the participation of the dorsal pathway in auditory LTM. We examined the effects of applying repetitive TMS (rTMS) to the pSTG and the IFG in separate experiments while participants listened to a list of pseudowords that they were asked to remember, as these were to be presented again later in a recognition memory test. As a control intervention in the recognition of pseudowords, we also applied rTMS to a nonspeech site in each of the two experiments. In contrast to most earlier rTMS studies, which used words, we chose pseudowords in order to avoid any semantic encoding, and we blocked working memory during the retention period so that participants could not use articulatory rehearsal, a working memory related process, to recognize the pseudowords. Articulatory suppression (i.e., suppression of both overt and covert movement of the articulators for example [10,11,30–33] can interrupt the maintenance and rehearsal of stored material in the articulatory or phonological loop [8,34]. By instructing the participants to count tones that were presented in the retention period, and thereby engaging their phonological loop, we forced the participants to rely on auditory long-term memory to perform the recognition task.

## Methods

### Participants

Thirty healthy volunteers, all right-handed and native English speakers, were recruited for the study. They were divided into two groups of 15 participants each. One group (mean age, 26.6 ± 6.3 years; 9 females) was assigned to the pSTG experiment, and the other (mean age, 30.6 ± 9.2 years; 9 females) was assigned to the IFG experiment. One member of each group had to be excluded due to technical difficulties during TMS stimulation, and one member of the IFG group withdrew during the first experimental session. One further participant was removed from the IFG group due to difficulties performing the task (for details see section on statistical analysis).more than 8 errors during control stimulation. This performance cutoff was defined as a error rate during control stimulation that was more than 3 standard deviations above the mean detected during piloting. The results reported below are thus based on 14 participants in the pSTG experiment and 12 participants in the IFG experiment. Both experiments were approved by the Neuroscience Institutional Review Board (IRB) of the National Institutes of Health (NIH, 11-N-018). Before the experiments, all participants gave their informed oral and written consent in accordance with the Code of Ethics of the World Medical Association (Declaration of Helsinki) and the NIH guidelines. Prior to participation all participants completed a neurological examination, standard at the National Institute of Neurological Disorders and Stroke that included a gross hearing assessment. However no quantitative hearing exam was performed.

### Experimental Procedure

Participants were seated in front of a PC laptop (Dell Latitude D610) and fitted with foam insert headphones (Etymotic Research, Elk Grove, Illinois). The laptop was used to present the stimuli and record the behavioral responses, and the headphones ensured stimulus clarity and sound-protection during rTMS.

**Fig 1:**
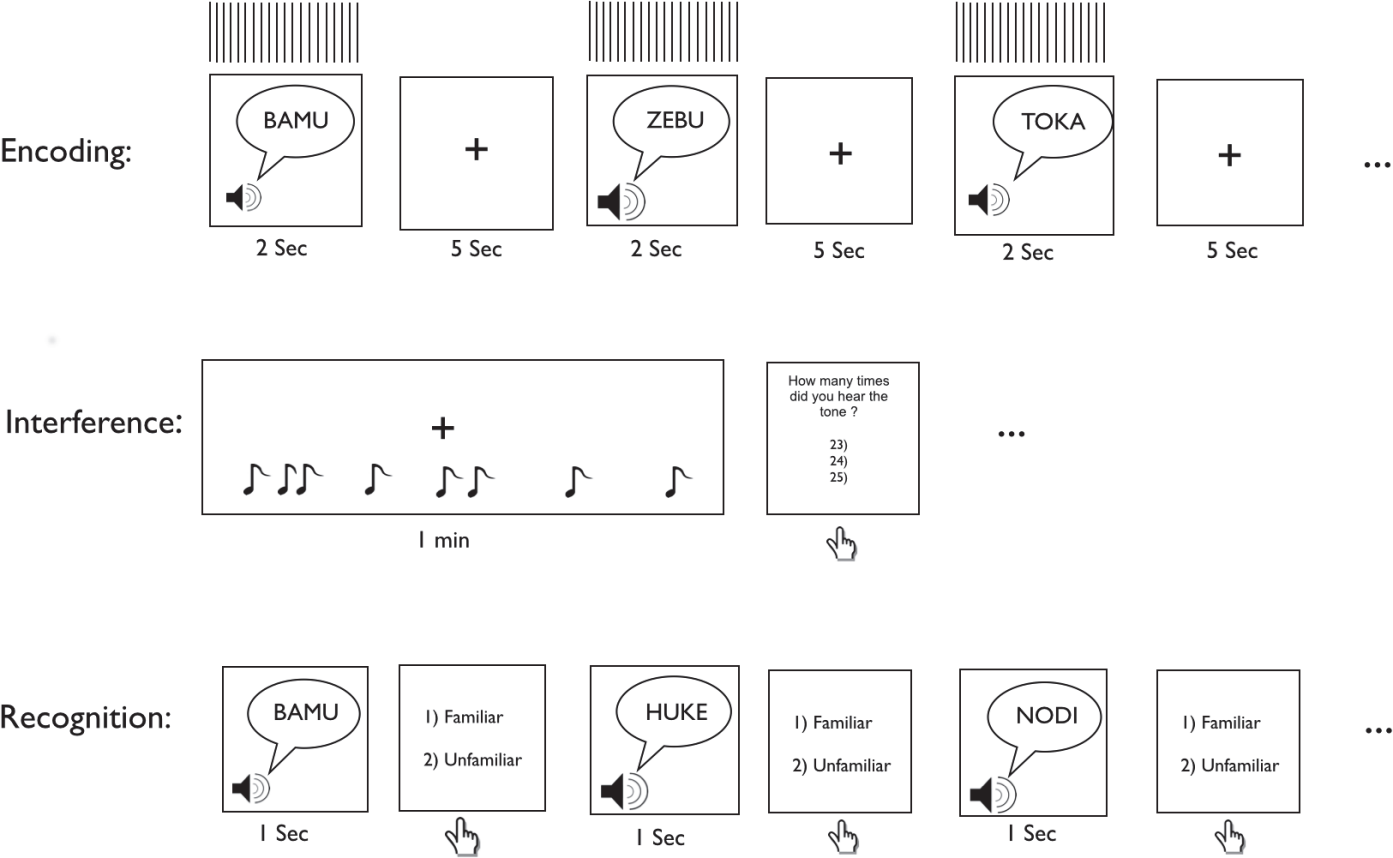
Illustration of the study design.

**Fig 2:**
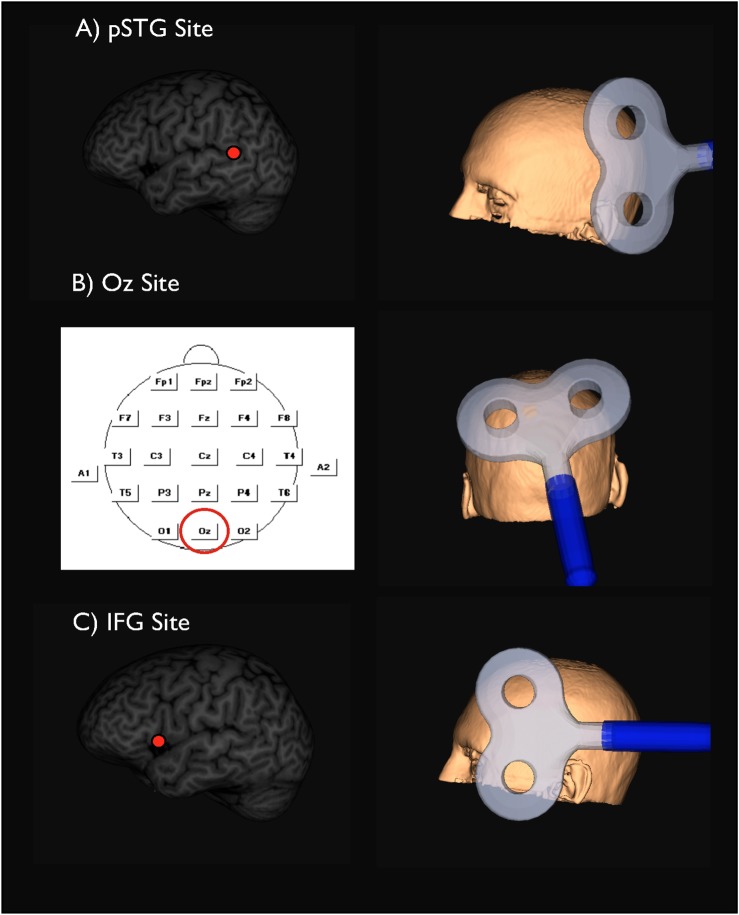
The mean location of the target and orientation of the stimulator during rTMS of: (A) caudalmost portion of the temporal gyrus, pSTG; (B) control site, Oz; and (C) inferior frontal gyrus, IFG.

Auditory long-term memory was tested in two sessions, each consisting of an encoding, interference, and recognition phase (Fig. 1). During encoding, the participant listened to one of two lists of 10 pseudowords, each pseudoword enveloped by 2 seconds of 10-Hz rTMS. The stimulation started 500 ms before pseudoword onset and ended 750 ms after pseudoword offset, the pseudoword itself also lasting 750 ms. The participant was asked to fixate a cross on the computer screen throughout this 2-sec period. The interval between pseudowords, and hence between rTMS pulses, was 5 seconds. In the first experiment, rTMS was delivered over the left Sylvian-parietal-temporal area (pSTG; active site; Fig. 2A) in one session and over the occiput (Oz; control site; Fig. 2B) in the other. In the second experiment, stimulation was delivered over the left inferior frontal gyrus (IFG; active site; Fig. 2C) in one session and over the Oz control site in the other. In each experiment, both the order of stimulation site (active site first, control site first) and the list order (list 1 first, list 2 first) were randomized across participants.

Prior to presentation of the pseudowords, the participants were instructed to try to remember the upcoming auditory stimuli without using elaborate memorization strategies (e.g., method of loci [35]). Immediately after the encoding phase, the participants were presented with a 5-minute articulatory interference task designed to prevent them from silently rehearsing the pseudowords they had heard in the encoding phase. This interference task required counting the number of times a randomly occurring tone (220 Hz) was presented via the headphones. After each minute of counting, the participants were asked to report, by pressing a button, the number of tones they had heard, with the tone occurring either 22, 23, or 24 times per minute; this procedure was repeated five times in quick succession while the participants continued to fixate the cross. Finally, during the recognition phase, which followed immediately after the interference task, 20 pseudowords were presented via the headphones. These pseudowords included the 10 that had been presented during encoding and 10 new ones. After each pseudoword, participants were instructed to indicate whether or not they had heard it before by pressing either the 1 key for ‘old’ or the 2 key for ‘new’. Immediately after each response choice, the next pseudoword was presented. Participants were instructed to keep their fingers at the ready on the response keys throughout this recognition phase. At the end of each session, participants were asked to rate the overall level of difficulty of the recognition task on a scale of 1–5 (1, extremely easy; 5, extremely hard. Participants were given a 1-hour rest period between the two rTMS sessions. Because the after-effects of rTMS are transient, a 1-hour rest interval is thought sufficient to avoid carry-over effects between the two stimulation sites [36].

In case a t-test indicated a significant group-level difference between control and active site, participants were invited back approximately one month after the main experiment. This was only the case in the pSTG group, so only the participants of this group were invited back for a perceptual control test. This was done in order to determine whether or not stimulation of the pSTG site during presentation of a pseudoword interfered with their perception of it, as measured by their ability to discriminate between pseudowords. For the perceptual control experiment, the participants were again instructed to fixate a cross on the computer screen while listening this time to 20 pairs of pseudowords separated by an intrapair interval of 750 ms. The first item in each pair was enveloped as before by 2 seconds of 10-Hz rTMS stimulation (with stimulation lasting until the end of the intra-pair interval of 750 ms), and, immediately after presentation of the second item in the pair, the participants were asked to judge whether the second item was the same as the first (by pressing the 1 key) or different from it (by pressing the 2 key). The pseudowords used in the control experiment were different from the pseudowords used in the main experiment but constructed on the same principles (See S1 Fig. for a visualization of the perceptual control task and some example stimuli). As in the recognition experiment, the pSTG site was stimulated in one session, and the Oz site, in a separate session. Again, session order was randomized and, to exclude carry-over effects, the two sessions were separated by a 1-hour rest period.

### Stimuli and Behavioral Measures

The pseudowords, which were easily mimicked but had no meaning, were each 4 letters and 2 syllables in length and 750 ms in duration. They were generated with a speech synthesizer using a UK English female voice (http://cepstral.com) and modified for length and loudness using Adobe Audition 3.0 (http://www.adobe.com/products/audition/). The sound intensities were adjusted by the experimenter to a level at which the participant could hear the pseudowords clearly during rTMS. As noted earlier, pseudowords were chosen as stimuli instead of real words to avoid semantic associations and thereby encourage stimulus-specific oromotor encoding and storage.

### rTMS

Repetitive TMS was produced by a MagStim Super-Rapid (MagStim, Whitland, Wales, UK) stimulator connected to a double 70-mm, air-cooled coil attached to a Numatic air blower (Numatic International, Chard, UK). An additional PC running Signal software and a Micro 1401 data acquisition unit (Cambridge Electronics Design, Cambridge, England) were used to trigger the magnetic stimulators that delivered the pulse trains. The timing of the pulse trains was synchronized with the behavioral tasks by interfacing the parallel port of the laptop computer with the trigger input of the Micro 1401 unit.

Before the two rTMS sessions, we measured each participant’s resting motor threshold of the right first dorsal interosseus muscle (RMTFDI). The active electrode was placed over the muscle belly, and the reference electrode, over the joint of the second finger. EMG was recorded using a Nicolet Viking IV biological system (Madison, Wisconsin, US). The RMTFDI was defined as the lowest intensity with which a single TMS pulse given over the cortical area M1 ‘hotspot’ for the FDI induced a motor evoked potential (MEP) of at least 50-μV peak-to peak amplitude in at least five out of ten trials.

During the encoding phase of the recognition task, the 10-Hz rTMS pulse train with which each pseudoword was paired was set at 110% of the participant’s RMT. However, if this RMT level was over 90% of the maximum stimulator output, as was the case for two participants in the pSTG experiment and one in the IFG experiment, the stimulation intensity was set at 100% of the RMT. The average stimulation intensity in the pSTG group was 64.6 (±11) percent of stimulator output and the average stimulation intensity in the IFG group was 65.4 (±10) percent of stimulator output.

The temporo-parietal site was defined as the caudalmost portion of the superior temporal gyrus (pSTG; BA 22) and the IFG site was defined as the ventralmost portion of the pars opercularis (vpPO; BA 44). We chose individual MRI-guided TMS neuronavigation since this technique has been shown to be superior to both functional Talairach coordinates and 10–20 EEG positioning [37]. The coordinates for pSTG were x = -57 ± 3.9; y = -50 ± 7.3; z = 18 ± 5.9 (MNI, mean ± sd), and those for IFG were x = -48 ± 3.6; y = 15.3 ± 2.8; z = 2 ± 2.7 (mean ± sd). The Oz control site in both experiments was determined according to the 10–20 EEG measurement system, which defines Oz as lying above the inion by 10% of the distance along the participant’s nasion-inion line. For precise positioning of the hand-held coils over both the experimental and control sites, we used Brainsight Neuronavigation (Rogue Research, Montreal Quebec, Canada) and magnetic resonance imaging (MRI), with each individual participant’s scan normalized a posteriori to the Montreal Neurological Institute (MNI) brain template. (Prior to the present experiments, we tested 13 other participants using procedures identical to those described here, except that the 10-Hz rTMS train applied to IFG was set at 100% instead of 110% RMT; see S2 Fig. and S1 Methods.)

### The Drift Diffusion Model

To address that we observed differences in accuracy without an accompanying drop in reaction time and to estimate cognitive processes underlying task performance we used a simplified diffusion drift model (EZ-diffusion model [38]). This simplified version allowed us to calculate the three most important unobserved variables of a two-choice decision task: (1) The decision boundary a, interpreted as a measure of response conservatism with large values indicating that the process takes more time to reach the boundary (2) The drift rate v, indicating the mean approach rate to the decision boundary. It is interpreted as the relative amount of information that is absorbed per time unit and indicates task difficulty. (3) The non-decision time T_er, summarizing all non-decision constants like the motor response. The simplified model used here, does not allow modelling RT distributions or to separately estimating the parameters for correct and erroneous trials, however, due to the limited amount of data collected in this experiment a more ‘macroscopic’ modeling approach was the only available option. The EZ-diffusion model (open-source matlab implementation: http://www.ejwagenmakers.com/papers.html) calculates v, a and T-er by taking the mean response time, the variance of response time, and response accuracy as input.

### Statistical Analysis

Behavioral data were processed in Excel, and all data were checked for normality distribution using the Kolmogorov-Smirnov test. For each dependent measure (recognition accuracy, reaction time, task difficulty as well as false alarm, miss rate and EZ-diffusion parameters) a repeated-measure ANOVA including the between-subject factor Group (pSTG vs. IFGBroca) and the within-subject factor Stimulation Site (experimental vs. control) was used. In case of a significant main effect or interaction effect post-hoc Tukey tests were applied.

Unpublished pilot data on stimulus validity suggested average error rates around 20% (mean: 4 errors ± 1.4) for our pseudo-word lists. To ensure all participants did perform the task reasonably well, we aimed at removing participants whose baseline performance exceeded an error rate of 40%. This level was determined by the mean error rate from the behavioral pilot plus three standard derivations. All analyses were carried out using Statistica 9.1 (Statsoft, Inc., Tulsa, OK, USA).

## Results

### Basic Performance Measures

**Fig 3:**
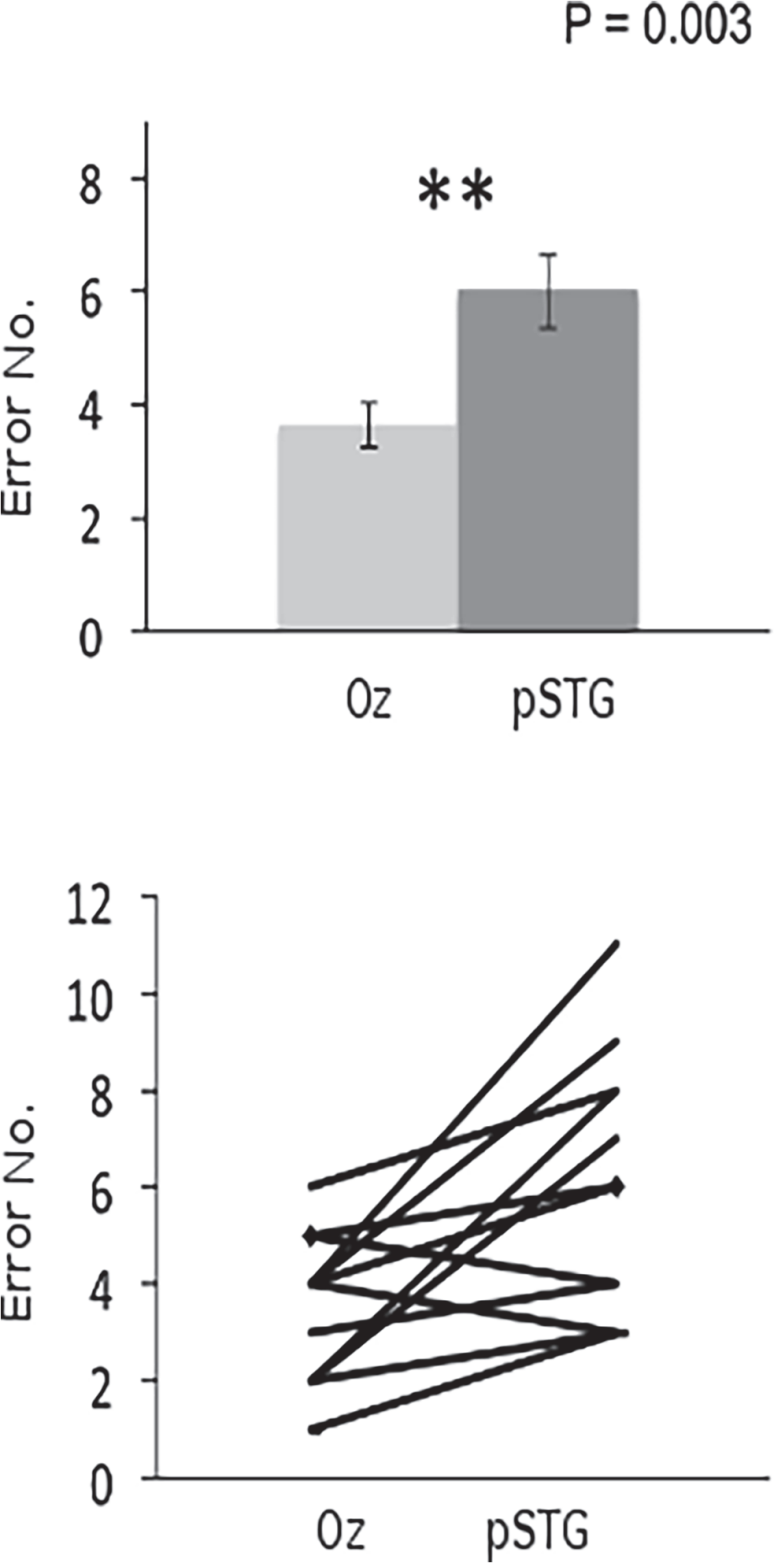
Upper graph: Recognition errors (group mean +/- SE following stimulation of Oz (control site) and pSTG (experimental site). Lower graph: Each participant's recognition errors following stimulation of Oz and pSTG. The line marked by diamond end-points represents the performance of three participants with the same scores.

**Fig 4:**
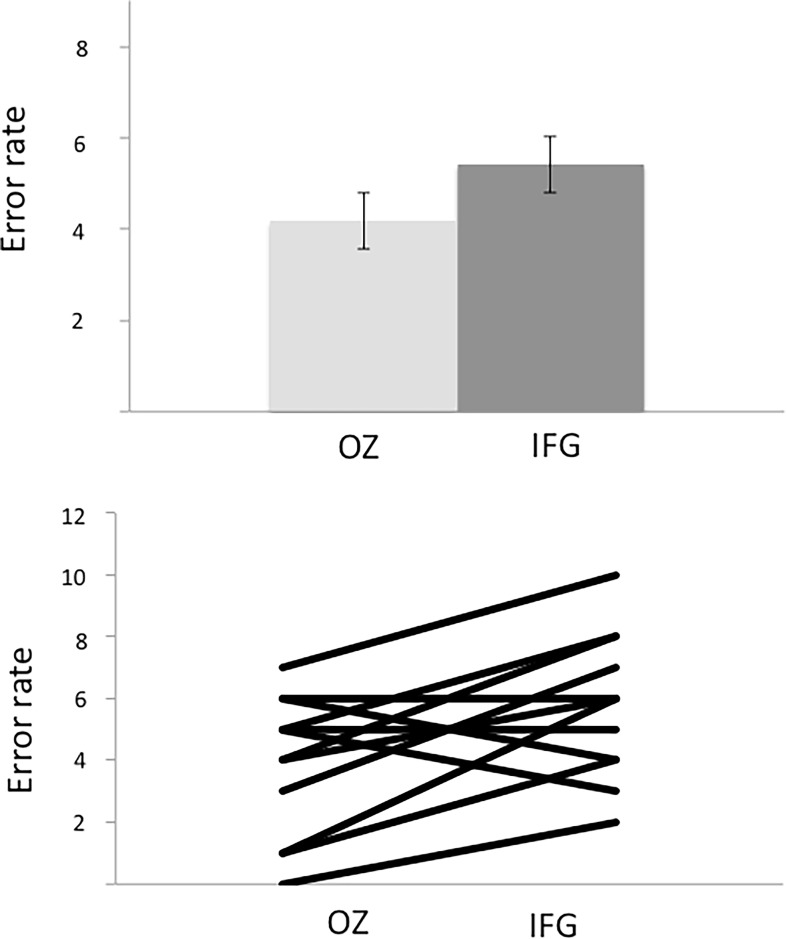
Upper graph: Recognition errors (group mean +/- SE following stimulation of Oz (control site) and IFG (experimental site). Lower graph: Each participant's recognition errors following stimulation of Oz and IFG.

The data were normally distributed according to the Kolmogorov-Smirnov test. Table 1 shows the descriptive statistics for the basic performance measures (error rate, reaction time, difficulty rating). For the error rates, the within-subject factor Stimulation Site (active vs. control) showed a significant effect (F(1) = 12.81, p = 0.001), with the error rate being higher in the active than in the control condition across groups. Neither the main effect for the between-subject factor Group or the Group x Stimulation Site interaction was significant (F(1) = 0.001, p = 0.96 and F(1) = 1.21, p = 0.282, respectively). Direct comparisons of Stimulation Site showed that the effect was heavily driven by a significant difference between active and control stimulation in the pSTG group (p = 0.01), whereas the post-hoc comparison between active and control in the IFG group did not reach significance (p = 0.35) (Figs. 3–4).

**Table 1:** Descriptive statistics for the basic performance measures.

Basic measures	Active			Control		
	Error	RT (ms)	Rating	Error	RT (ms)	Rating
pSTG Experiment	6.0 (±2.4)	949 (±310)	3.4 (±0.8)	3.6 (±1.4)	861 (±348)	3.1 (±0.5)
IFG Experiment	5.4 (±2.3)	781 (±345)	4.0 (±0.8)	4.1 (±2.3)	829 (±284)	3.5 (±0.6)

For the reaction times the rmANOVA did not show any significant main effects or interactions (Group: F(1) = 0.081, p = 0.37, Stimulation Site n: F(1) = 0.098, p = 0.76, and Group x Stimulation Site n F(1) = 1.14, p = 0.294).

For the difficulty judgments the rmANOVA did show a significant effect of Group F(1) = 5.91, p = 0.022 with the pSTG group rating both tasks as easier than the IFG group, however, there was no significant Stimulation Site effect or a Stimulation x Group Interaction (F(1) = 2,62, p = 0.11, and F(1) = 0.16, p = 0.68, respectively).

### Perceptual Control Task

Since only the pSTG group showed a significant within group difference between active and control stimulation we called these participants back for a perceptual control task to test if the difference was caused by sensory deficits. Here, a student’s t-test was used to compare the effect of experimental and control site. The perceptual control experiment failed to differentiate between the effects of stimulating the experimental and control sites, as under both conditions all participants discriminated between the two members of each of the 20 pairs of pseudowords with 100 percent accuracy. There was also no detectable difference in the reaction times (experimental, 707 ms ±347; control, 687 ms ±292; t[12] = 0.43, p = 0.67) or in the mean perceived difficulty of the control task (experimental, 1.4 ± 0.7; control, 1.2± 0.5; (t[12]) = 1.00; p = 0.34).

### Diffusion Model Parameters

Table 2 shows the descriptive statistics for the diffusion model parameters (v, a, T_er). An rmANOVA using the drift rate v (measure of task difficulty) as the dependent variable showed a significant effect of Stimulation Site (F(1) = 10,94; p = 0.003), with lower drift rates (i.e., higher task difficulty) when stimulating the active site. Also for v there was no significant Group effect (F(1) = 0,72; p = 0.402) or Group x Stimulation interaction (F(1) = 1,57; p = 0.222) but the post-hoc tests showed again that the main effect of Stimulation Site was strongly driven by the effect in the pSTG group (p = 0.01), in the IFG group post-hoc tests did not show significant differences between active and rest (p = 0.52). For the parameters indicating response conservativeness and non-decision time (a and T_er) neither main effects (all p-values > 0.4) nor interactions were significant.

**Table 2:** Descriptive statistics for the diffusion model parameters.

EZ-diffusion model	Active			Control		
	v	a	T_er	v	a	T_er
pSTG Experiment	0.03 (±0.02)	0.25 (±0.07)	-0.7 (±0.7)	0.07 (±0.02)	0.25 (±0.05)	-0.59 (±0.4)
IFG Experiment	0.03 (±0.02)	0.23 (±0.09)	-0.72 (±0.7)	0.05 (±0.03)	0.26 (±0.07)	-0.81 (±0.6)

### Error Types

**Fig 5:**
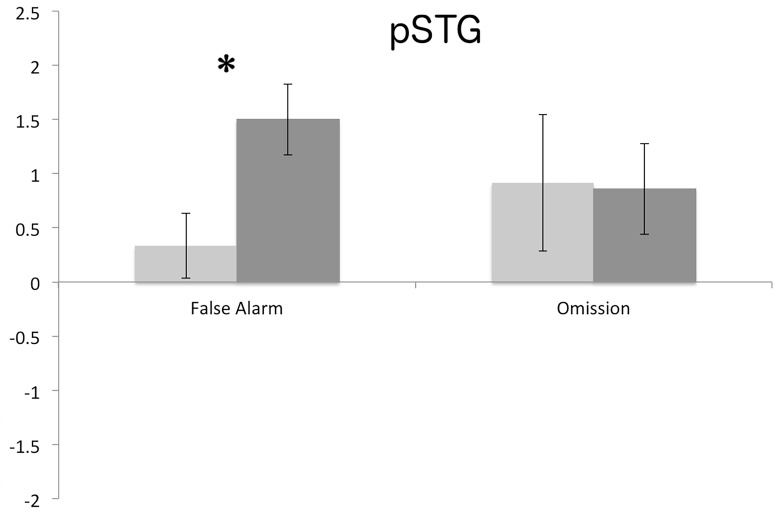
Normalized false alarm and omission errors (group mean +/- SE) for IFG and pSTG stimulation.

Table 3 shows the descriptive statistics for the different error types (False alarm, Miss). Since our simplified diffusion model did not allow for a detailed modeling of specific error responses, we investigated the effect of stimulation on false alarms (i.e., judging a new pseudoword as old) and misses (i.e., judging an old pseudo word as new) separately. The rmANOVA for false alarms did show a significant main effect for Stimulation Site (F(1) = 28,40, p < 0.001) as well as a significant Group x Stimulation interaction (F(1) = 9,11, p = 0.006). Post-hoc tests again confirmed that only the pSTG group showed significant differences in false recognition rate. In the pSTG group false alarms were higher following active compared to control stimulation (p < 0.001) (Fig. 5). For the misses, no significant main effect or interaction could be found (Group: F(1) = 0.30, p = 0.585, Stimulation Site: F(1) = 3.95, p = 0.058, and Group x Stimulation F(1) = 0.19, p = 0.662).

**Table 3:** Descriptive statistics for the different error types.

Error types	Active		Control	
	False alarm	Omission	False alarm	Omission
pSTG Experiment	2.5 (± 1.5)	3.6 (± 1.4)	0.9 (± 1.0)	2.6 (± 1.4)
IFG Experiment	1.9 (± 1.3)	4.0 (± 2.1)	1.4 (± 2.3)	3.4 (± 2.0)

## Discussion

Of the two speech sites we stimulated, the posterior portion of the superior temporal gyrus (pSTG) and the inferior frontal gyrus (IFG), our results provide clear support for a role in auditory long-term memory of only the first of these. Thus, compared to the mnemonic effects of stimulating a nonspeech control site, rTMS applied to the left pSTG during pseudoword encoding resulted, as predicted, in a highly significant impairment in the participants' ability to recognize those pseudowords after a 5-min delay interval filled with an articulatory suppression task. That this stimulation-induced deficit was not attributable to interference with the participants' auditory perception was demonstrated by their preserved ability to discriminate between two matched and two nonmatched pseudowords when the members of each pair were presented in rapid succession. By contrast to the unambiguous mnemonic effect of stimulating the pSTG, stimulation of the IFG led to only a weak, nonsignificant trend toward recognition impairment. Additional analysis using a simplified drift model indicatedindicates that the parameter v, indicating task difficulty, during encoding was significantly affected by stimulation over the active sites. As for the error rate, rTMS applied to the left pSTG during pseudoword encoding resulted in significantly decreased drift rates, indicating a decrease in perceived signal quality during this condition. Even though IGF stimulation also led to a decrease in drift rate, the within group difference for the IFG group was non-significant. A similar pattern could also be observed for the false alarm rate (i.e., identifying a new word as old) where false alarms were significantly higher only after pSTG simulation. The different outcomes following stimulation of the two different experimental sites will be considered in turn.

As summarized in the Introduction, left temporo-parietal areas in and around the pSTG [39] are critical for the transposition of speech sounds into oromotor sequences and have been implicated in verbal working memory [22]. Additionally, lesions that include the pSTG cause severe impairment in verbal working memory [39].

Given the evidence that working memory ability depends on the activation of stimulus representations stored in long-term memory [40,41] the impairment reported here in long-term memory is consistent both with the literature on working memory cited above and with the impairment others have observed in working memory following rTMS applied to a slightly more anterior temporo-parietal site (e.g., reduced digit span [28] and reduced active pseudoword recall [42]).

Our results also showed that, pSTG stimulation specifically affected encoding since the drift rate was the only diffusion model parameter significantly affected by pSTG stimulation. The fact that neither the decision boundary nor the non-decision time was significantly affected indicates that pSTG stimulation selectively disrupted encoding without interfering with non-specific retrieval processes or a general tendency to change the decision boundary during retrieval.

When comparing error types, pSTG stimulation increased false alarm rates (FA) while misses stayed unaffected. This complements the findings of the drift model since it is known that higher task difficulty can cause participants to commit more false alarm errors [43–45]. Taken together, the results thus suggest that only pSTG stimulation led to a shallow encoding of pseudowords, thereby prompting participants to more false alarms.

Our combined evidence suggests that the pSTG serves as a gateway between the auditory and oromotor systems, and, in the process, transposes unfamiliar speech sounds into oromotor representations that are encoded and stored long-term, thereby leading to the strong memory traces for those sounds.

The companion hypothesis that the left IFG is also an essential site for encoding and storing the motor representations of speech sounds was not supported by the present results. A negative outcome of applying rTMS to this site was unexpected, inasmuch as previous studies [27,28,46] had reported that stimulation of the left IFG interferes with short-term phonological memory. However, the nonsignificant trend toward decreased recognition accuracy that we observed after IFG stimulation should probably not be dismissed. One possible interpretation is that the trend signals a genuine though weak memory impairment caused by spread of the IFG stimulation caudally from its focal point in the ventral IFG to the ventral premotor cortex, which some investigators [28,47,48] have proposed is the more critical locus for encoding the memory of speech sounds. Indeed, our IFG stimulation site (x = -48, y = 15, z = 0) lay rostral to the stimulation site reported by Romero and colleagues [28] (x = -46, y = 2, z = 16). However, in our data set we could not find a systematic trend that participants who showed memory impairment after IFG stimulation had a more rostral stimulation site than participants who showed no change or an improvement (impaired: x = -47, y = 15, z = 0 vs. same/improved: x = -49, y = 15, z = 0). Another possibility is that rTMS over the IFG merely led to incidental stimulation of the facial muscles around the eye and jaw (see Fig. 2), which were sometimes observed to twitch slightly, thereby possibly causing sporadic inattention. Since facial muscle stimulation was not an issue during control stimulation over the occipital cortex, the sporadic inattention it caused might be a reason for the non-significant increase in error rate. These issues need to be resolved before it will be possible to determine from rTMS evidence whether or not the IFG plays an essential role in long-term auditory memory.

There are some caveats that should be discussed in connection with both the IFG and the pSTG experiment:

First, we used a relatively small number of stimuli in the experiment. We decided on a small number of pseudowords per condition since, considering the high mean baseline–error rates of 20%, an increased number of stimuli would have likely meant that some participants would not exceed chance performance in the control condition. The small number of stimuli meant however, that the absolute change in performance was relatively small. The number of stimuli also meant that our study had relatively low power. For the detected difference following pSTG stimulation this has no direct implications since a small sample size does not affect a type I error but it may have prevented the detection of more subtle changes following IFG stimulation. However, the IFG results presented here are replicated by the data in the supplementary material where IFG stimulation in the same experimental setup was given at an only10% lower intensity. The results from this additional experimental group strengthen the reliability of our IFG finding.

Finally, that active and control TMS were applied on the same day might have an additional caveat. We cannot completely exclude the possibility of carry-over effects, even though the literature suggests that this was not the case, since the excitability increasing effects of short high-frequency rTMS trains usually do not outlast the stimulation by more than a couple of minutes [36,49].

## Supporting Information

S1 Fig Illustration of the perceptual control task. (PDF)

S2 Fig Upper graph: Recognition errors (group mean +/- SE following stimulation of Oz (control site) and IFG (experimental site) at 100% RMT. Lower graph: Each participant's recognition errors following stimulation of Oz and IFG at 100% RMT.
(PDF)

S1 Methods Supplementary information concerning the additional group where IFG and OZ were stimulated at 100%RMT. (DOCX)
