# Supplementary figures and images for: Participation of the Classical Speech Areas in Auditory Long-Term Memory
Source: PLoS One. 2015 Mar 27;10(3):e0119472. doi: 10.1371/journal.pone.0119472 (PMC4376917; doi:10.1371/journal.pone.0119472)

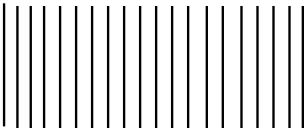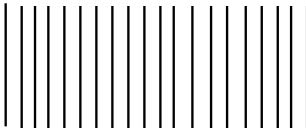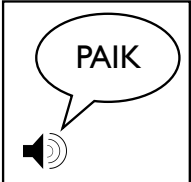

+

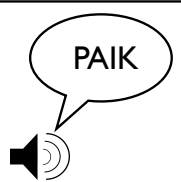

- 1) Same
- 2) Different

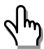

+

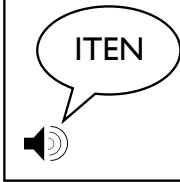

+

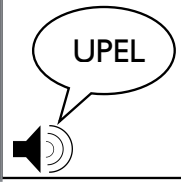

- 1) Same
- 2) Different

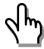

...

Perceptual

1000 ms

750 ms

1000 ms

1000 ms

750 ms

1000 ms

Supplement: S1 Fig — (PDF) [file pone.0119472.s001.pdf]

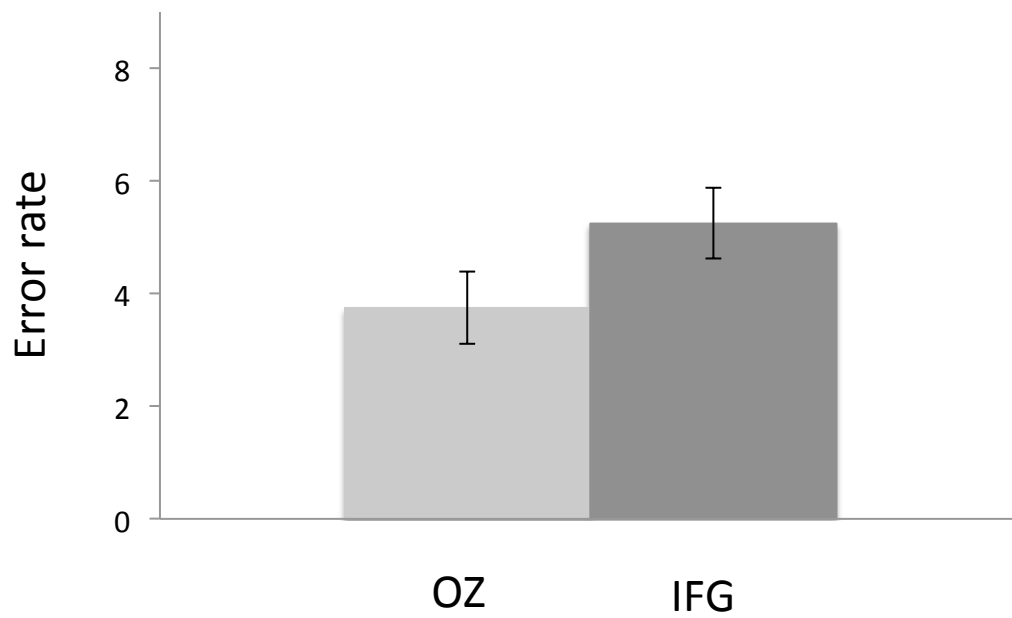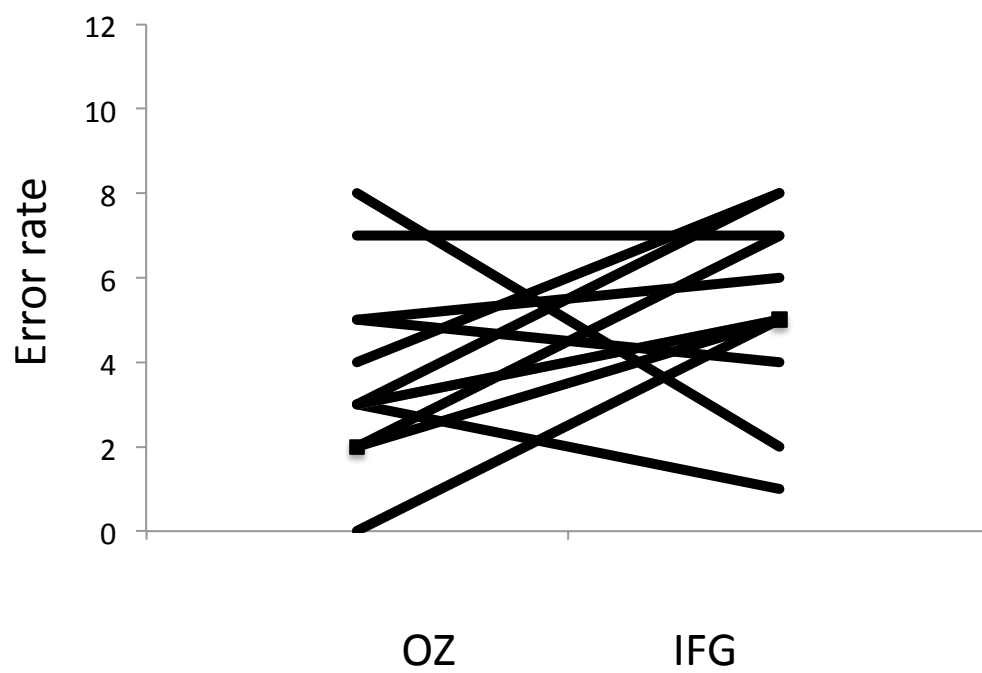

Supplement: S2 Fig — Lower graph: Each participant's recognition errors following stimulation of Oz and IFG at 100% RMT. (PDF) [file pone.0119472.s002.pdf]
