# Supplementary material for: Participation of the Classical Speech Areas in Auditory Long-Term Memory
Source: PLoS One. 2015 Mar 27;10(3):e0119472. doi: 10.1371/journal.pone.0119472 (PMC4376917; doi:10.1371/journal.pone.0119472)
Supplement: S1 Methods — (DOCX) [file pone.0119472.s003.docx]

Supplementary Material:

12 participants were tested in the same experimental setup and using the same statistical tests, when an rTMS train was set 100% RMT. IFG coordinates were: x = -47.5 ± 1.7; y = 13.3 ± 2.9; z = 7.1 ± 2.8 (mean ± sd). One participant was excluded from further analysis since his error rate during control stimulation exceeded our threshold of 8 errors.

Results:

*IFG - 100% experiment*

The data were normally distributed according to the Kolmogorov-Smirnov test. After IFG stimulation, participants averaged 5.2 recognition errors (±2.2) compared with 3.7 errors (±2.2) after control stimulation (*t*_(11)_ = -1.55; *p* = 0.14). Neither mean reaction time for the recognition judgments (IFG, 955 ms ±286; control, 995 ms ±356; *t*_[11]_ = 0.30, *p* = 0.76) nor mean perceived difficulty of the tasks (IFG, 3.5 ± 1.2; control, 2.9± 1.2; (*t*_[11]_) = -1.98; *p* =0.07) differed significantly between the experimental and control stimulation sites. The supplementary figure shows the mean and single subject error rates.
